# Are exergames promoting mobility an attractive alternative to conventional self-regulated exercises for elderly people in a rehabilitation setting? Study protocol of a randomized controlled trial

**DOI:** 10.1186/s12877-015-0106-0

**Published:** 2015-09-07

**Authors:** Viviane Hasselmann, Peter Oesch, Luis Fernandez-Luque, Stefan Bachmann

**Affiliations:** Rehabilitationsklinik Walenstadtberg, Walenstadtberg, Switzerland; Rehabilitationsklinik Valens, 7317 Valens, Switzerland; University of Seville, Seville, Spain; Norut, Tromsø, Norway

## Abstract

**Background:**

Maintaining mobility in elderly persons has become a primary goal within healthcare services. In older adults, exercise programs significantly reduce the risk of falling and death. Long-lasting and high-intensive multi-component exercises are most effective. In a rehabilitation setting, self-regulated exercises are conventionally taught by physiotherapists, using handouts. However, the adherence of elderly persons to executing these self-administered programs varies considerably. They are often considered tedious and boring, and thus prematurely stopped.

The primary aim of this clinical trial is to determine whether elderly persons in a rehabilitation setting show higher adherence to self-regulated training when using exergames than when performing conventional exercises. The second objective is to explore which mode of exercise leads to greater improvement in balance performance.

**Methods/Design:**

The study consists of a single blind, stratified, randomized control trial with two parallel groups. Once included, study participants will be stratified according to their balance and computer skills and randomly allocated to self-regulated training with conventional exercise programs or with exergames played with the Windows Kinect® sensor and FitBit® pedometer. In both groups, self-administered exercise programs will be taught by experienced physiotherapists and performed at the patient’s own discretion during the ten days of intervention. The primary outcome is the performed daily training volume, collected by the participants in a logbook. Secondary outcomes are objective and subjective balance skills measured by an activity tracker and the Fall Efficacy Scale self-administered questionnaire. Both assessments will be performed at pre- and post-intervention.

**Discussion:**

According to the available literature, this study is the first to compare conventional self-regulated exercises with exergames among older patients in a rehabilitation setting. Results of this study will contribute to our understanding of its motivational potential on exercise adherence in elderly persons and provide more insight into the potential effectiveness of exergames promoting mobility.

**Trial registration:**

The present clinical study has been registered on ClinicalTrials.gov under the identifier number: NCT02077049. The detailed trial protocol can be accessed online on: NCT02077049.

**Electronic supplementary material:**

The online version of this article (doi:10.1186/s12877-015-0106-0) contains supplementary material, which is available to authorized users.

## Background

European society is experiencing a significantly increased proportion of persons over 65 years of age [1, 2]. Ageing is accompanied by a decline in mental functions leading to a reduced motivation for physical activities, as well as a decline in motor skills resulting in mobility impairment and higher risk of falling [3, 4]. However it has been proven that increased physical activity and training intensity help to maintain independence in daily living activities and mobility, thus reducing the risk of falling and consequently lower institutional placement and mortality [5]. In this regard, the World Health Organization (WHO) recommends persons over 65 should practice aerobic physical activity for at least 150 min of moderate intensity or 75 min of high intensity per week [6]. Moreover, elderly persons should perform strengthening exercises at least twice a week and balance exercises at least three times a week [7]. In a rehabilitation setting, exercise programs are also planned and structured in order to achieve these WHO recommendations. In order to increase the quantity and duration of therapy offered to patients, they are therefore prescribed self-regulated exercises. In other words, in addition to therapist-supervised sessions, in-patients are encouraged to train by themselves without closed supervision active exercises promoting mobility, balance and muscle strengthening.

However, the compliance of the elderly to execute such physical activities by themselves varies considerably. These conventional exercise programs are often considered tedious and boring, hence prematurely stopped [8–11]. An attractive alternative to increase the elderlies’ motivation for self-regulated exercises are exergames. Exergames are a subtype of serious games, i.e., designed for a primary purpose other than pure entertainment, but the user has to perform physical exercises to control the game. They rely on technology that tracks body movement and reaction, and are designed to promote an active lifestyle by using persuasive technology [12].

Nowadays exergames are available on the market in the leisure sector and are increasingly used in rehabilitation settings [13]. It has been demonstrated that the risk of falling can be reduced by the use of low-cost commercially available tools, such as Nintendo® Wii [14–16]. However, elderly users are often not familiar with computer technology. They therefore frequently experience constraints and difficulties in the execution of exergames from the leisure sector [16–19].

To improve the usability of exergames for the elderly, simple startup and menu navigation are required [18, 20]. In addition, adjustable navigation speeds and a pause option to interrupt and restart the game at any time further facilitate the use of exergames for older players. But above all, for geriatric rehabilitation purposes these games must be task-oriented and closely map real world activities [21, 22].

Older user-friendly exergames combined with game-based extrinsic motivation (such as instant feedback, social play, personalization and persuasive technologies) have an enormous potential to motivate and encourage elderly persons to change their sedentary lifestyle and become more physically active in daily life as well as in performing exercises [23, 24]. The GameUp project addresses these specific challenges by developing new exergames promoting mobility in the elderly. Effective technologies are used for modifying behaviors such as persuasive technologies, physical exercises for balance, strength and endurance and social elements [25].

The primary aim of this clinical trial is to determine whether elderly patients in rehabilitative settings show higher adherence to self-regulated training when playing exergames than when performing conventional exercises. Secondly the study explores to what extent balance skills vary according to the mode of self-regulated training. The concept of balance is defined here as the objective balance skills of the patient in walking and his/her subjective fear of falling during various activities. The purpose of this paper is to describe the study protocol and to discuss challenges in the application of exergames with elderly users.

### Hypotheses

1. Elderly persons in rehabilitation settings will show higher adherence to self-regulated training when using exergames than when performing conventional exercises.
2. Exergames will lead to greater improvement in balance skills than conventional exercises.

## Methods

Our study protocol adheres to the CONSORT guidelines for clear, complete and transparent reporting [26].

### Study setting

It will be a single center clinical study performed in Switzerland at Walenstadtberg Rehabilitation Clinic. Most of its clientele are geriatric patients suffering from musculoskeletal impairment (i.e., from ortho-traumatology, internal medicine, oncology, pneumonology), and are referred from acute hospitals for inpatient post-operative rehabilitation purposes.

### Trial design

**Fig. 1:**
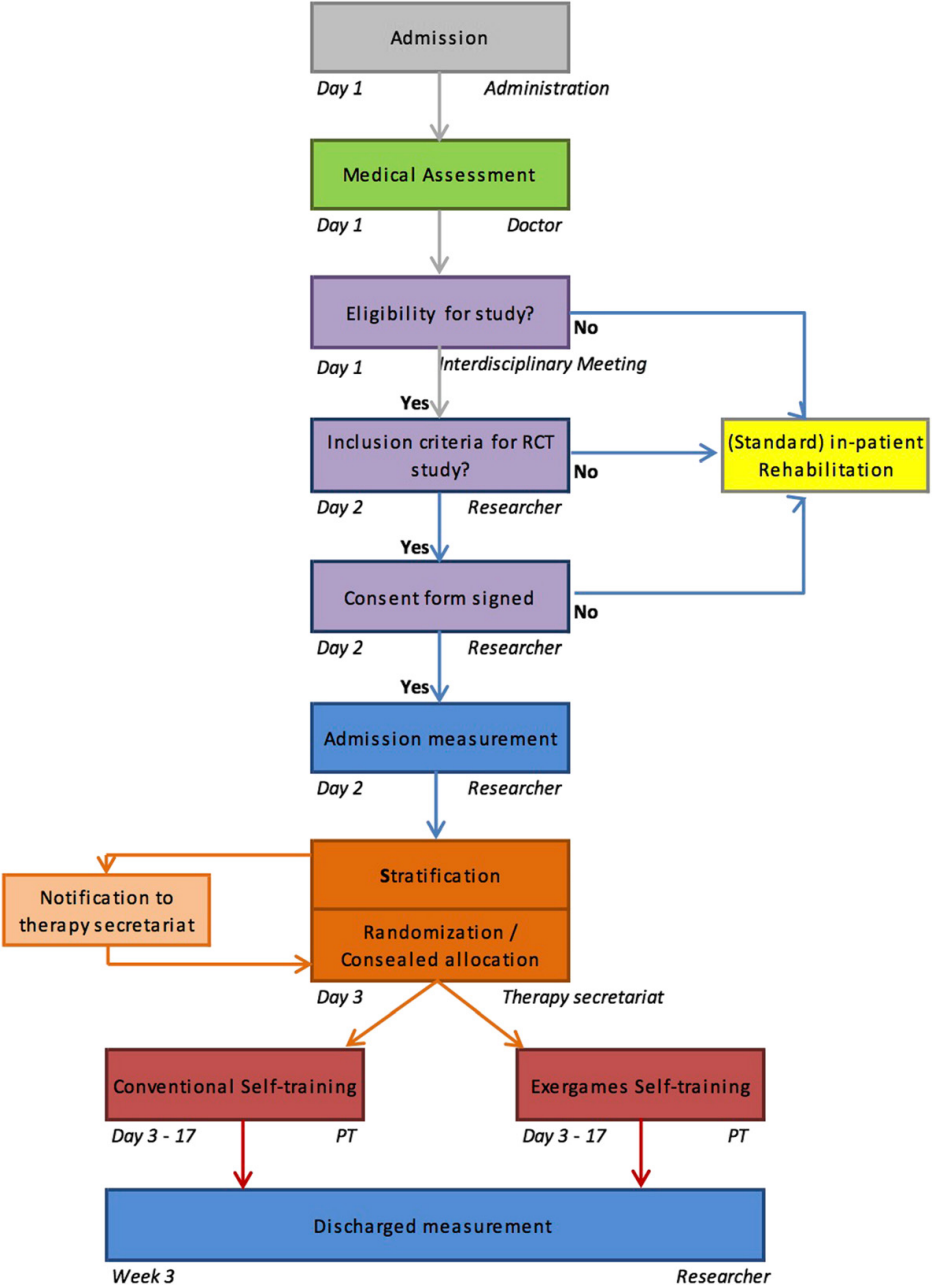
Study design. All patients referred to Walenstadtberg Rehabilitation Clinic are eligible for study inclusion. After medical screening by the doctor in charge, the interdisciplinary team composed of the doctor, the physiotherapist and the nurse in charged, considers the study eligibility of the patient. Whereupon the study examiner checks the patient for inclusion and exclusion criteria. Once the consent form is signed, participants are stratified into four groups according to their computer skills and their balance skills. An independent and blinded body (therapy secretariat) performs concealed randomization and allocation to either intervention or control group

The study will consist of a single blind, stratified, randomized controlled trial with two groups, parallel designs comparing self-regulated conventional exercises with exergames. Participants will be randomly allocated to the intervention group or to the control group, with an allocation ratio of 1:1. Study results will be collected daily during the intervention phase by the participants and at pre- and post-intervention by the study researcher. Figure 1 summarizes the study design. No important changes of methods have been done after trial start.

### Participants and recruitment

All patients referred for in-patient rehabilitation at Walenstadtberg Rehabilitation Clinic are eligible for study inclusion. After medical screening by the doctor and study eligibility by the interdisciplinary team (doctor, physiotherapist and nurse in charge), the patient will be checked for inclusion and exclusion criteria by the study researcher. Patients will be included in the study if they are above 65 years of age, are able to walk independently over 20 m (with or without walking aids), for whom self-regulated training has been prescribed. As a general rule in the clinic, a self-regulated conventional exercise program is prescribed to all post-operative patients (excepting specific medical contra-indications such as open wounds, severe pain, cognitive impairment…) with the objective of improving balance, strength and mobility.

Finally participants will have given a written informed consent. Patients presenting disorders limiting the use of computer games (e.g., neurological disorder, visual impairment, deafness, vertigo) or with cognitive impairment will be excluded. For the purpose of this study, cognitive impairment is defined as a Mini Mental State Examination (MMSE) score < 26 [27]. This cut off will also accurately detect cognitive dysfunctions in highly educated individuals [28].

### Interventions

The intervention trialed in this clinical study is a self-regulated exergames program among elderly people. Conventional self-regulated exercises as the control, are already part of the standard rehabilitation services prescribed at Walenstadtberg Clinic. Exercises in both groups (exergames or conventional) are similar and based on the same physiological assumptions and physical requirements of elderly people. Affordance levels of the exergames are therefore comparable to the conventional exercises.

**Fig. 2:**
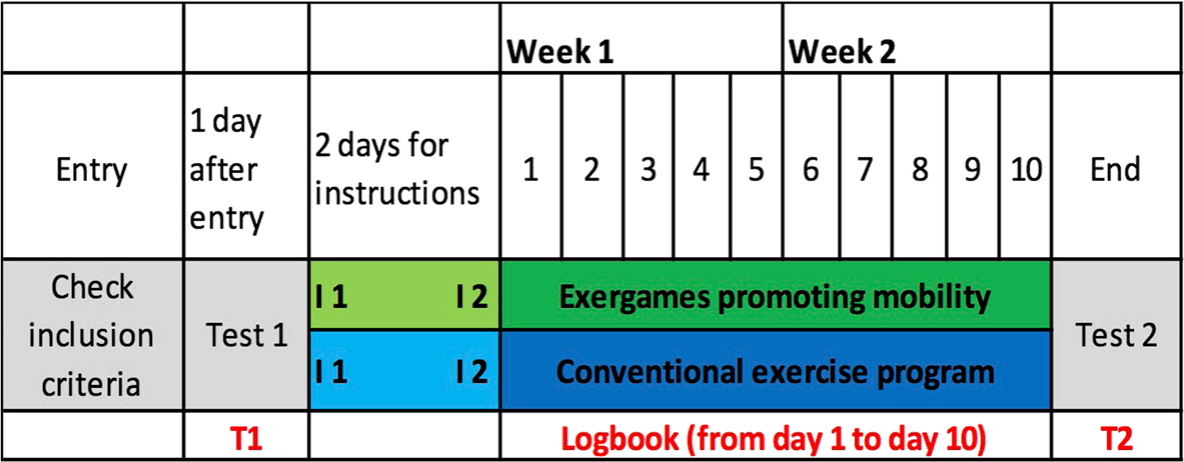
Study time flow. The researcher performs the subjective and objective balance tests (BBS, ActiGraph and FES-I) at the time point T1 (see Fig. 2) and grades the patient’s computer skills. Participants are then stratified into four groups and allocated randomly in the intervention group or the control group. During the two days preceding the start of the self-regulated training program (i.e., time points I1 and I2 in Fig. 2), the patient is instructed on how to perform the self-regulated exercise program according to his allocated group. The intervention period for the self-regulated training program lasts two weeks, namely 10 working days (from Monday to Friday each). On day 11 at the end of the intervention phase (time point T2), the subjective and objective balance skills are again tested by the researcher

All study participants will be entitled to two time-slots (2 × 30mins per day) from Monday to Friday, dedicated to self-regulated training for ten working days. This is communicated via the printed weekly therapy program containing the various medical appointments and therapy sessions of each day. Before the intervention starts, patients will receive two instruction sessions given by a trained physiotherapist on how to perform the self-regulated training (conventional or exergames). Additionally all patients are encouraged to walk and climb stairs instead of using the lift. This protocol ensures that all participants will receive the same attention at the beginning of the study and will be equally motivated to perform self-regulated training. Figure 2 shows the study time flow.

In order to ensure safety during self-regulated training, different levels of exercise difficulties in terms of balance and training duration for the conventional program as well as for the exergames were developed. Appropriate exercises will be selected by the physiotherapist according to the patient’s balance skills. The Berg Balance Scale (BBS) test will be used as a cut-off point. A BBS score < 45 indicates a risk of falling, and thus those patients will perform self-exercises in a sitting position only. Patients scoring between 45 and 55 points will perform the exercises in a static standing position, whereas patients reaching the maximum score of 56 will perform exercises in a dynamic standing position. The training program of both groups can be performed either sitting or standing (with or without support), making it accessible to all participants regardless of their balance abilities.

#### Exergames

**Fig. 3:**
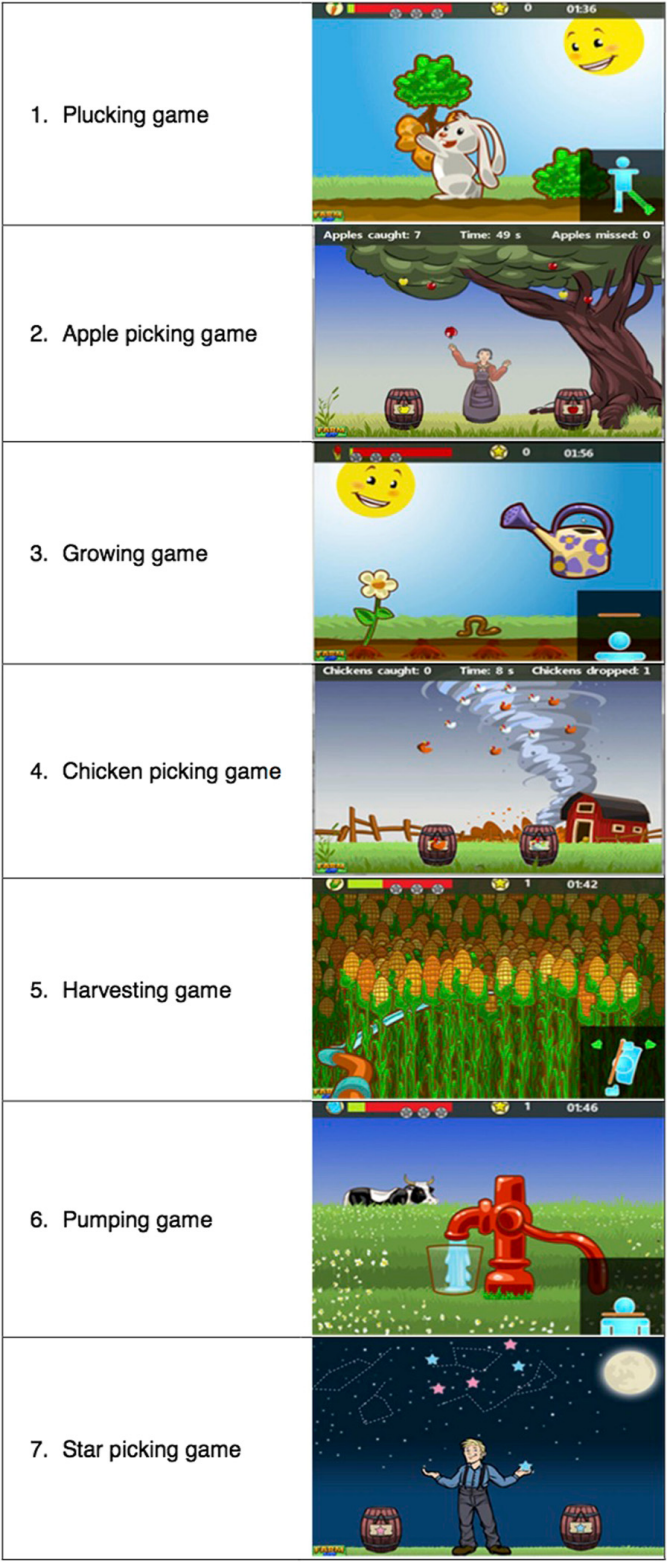
Pictures of GameUp exergames. The exergames consist of 7 mini-games, including balance, mobility, and strengthening exercises. Exercise 1 called “plucking game” is a strengthening exercise for abductor muscles. The patient has to spread apart one leg on the side. Exercise 2 “apple-picking game”, exercise 4 “chicken-picking game” and exercise 7 “star-picking game” are balance exercises where the patient has to catch the falling objects and put them in the correct receptacle. These three balance exercises are based on the same training principle where the patient has to move sideways. Exercise 3 called “growing game” is a strengthening exercise for the calf muscles. The patient has to stand on his toes to water the flower. Exercise 5 called “harvesting game” is a strengthening and mobility exercise for the trunk. It trains the torso rotation. The patient has to rotate his trunk in order to cut the corn with the scythe. Exercise 6 called “pumping game” is a strengthening exercise for quadriceps muscles where the patient has to perform squats in order to pump water into a glass

The exergames used in this trial were developed by the GameUp project, which is part of the Ambient Assisted Living Joint Programme Call 4 aiming for Information and Communication Technologies (ICT) based solutions for the advancement of older persons’ mobility [36]. Following WHO recommendations on physical activities and the usability requirements of older persons, the GameUp project created seven mini exergames training mobility, strength, and balance on Kinect® for Windows. However these exergames are currently not yet available to the public. Pictures of GameUp games are shown in Fig. 3. A One™ commercially available mobility tracker from the company Fitbit® will also be used for promoting endurance.

**Kinect®** is a motion sensing input device by Microsoft for Xbox® 360 video game consoles. Marketed since November 2010, it is meanwhile also available for Windows PCs allowing the development and programming of new game applications. The Kinect® device is made up of several video cameras and sensors specially adapted to track movements in a tri-dimensional space. It thereby enables users to control and interact with their screen without using a gamepad, but simply with an easy interface using gestures and spoken commands [37]. Thus, playing freely without being bonded to traditional gamepads makes the game console user-friendly and more suitable for older persons. Participants will train with the exergames in the therapy room where a TV and the Kinect® equipment is made available.

**Fitbit®**, with its integrated altimeter and tri-axial accelerometer accurately captures all daily activities. It tracks the number of steps taken, stairs climbed, distance traveled and calories burned every day [38]. Thanks to its low weight and small size, it can easily and discreetly be attached to clothing. The aim of this tracker is to empower and encourage the patient, by delivering real-time feedback that helps him/her be more active [39, 40].

#### Conventional exercises

Patients will perform the conventional exercises according to a printed instruction sheet (see Additional file 1). These self-regulated exercise programs are routinely offered as part of the standard rehabilitation services of Walenstadtberg Clinic. They consist of several active exercises either in sitting, standing or walking, depending on the patient’s balance skills and can either be performed in the patient’s room or in the therapy room.

### Outcome measures

#### Primary outcome

The primary outcome of this randomized controlled trial is the daily training volume of the self-regulated training performed individually. The daily training volume is operationalized by the frequency (quantity of sessions per day) and the total duration (time in minutes of all the performed sessions per day).

It was considered using the log data from the Kinect-based game and Fitbit® to measure daily training volume as well as steps performed. However, as these measurements were not possible in the control group it was decided to use a pro forma logbook in all participants. Patients recorded the number of sessions per day, how many minutes each session lasted and for how many minutes patients went walking.

Patients will also be asked to grade their motivation and enjoyment for each training day, according to a five-level Likert scale, rating respectively from *no motivation/fun* to *huge motivation/fun.* The feasibility of this logbook was tested in a pilot study comparing conventional balance training to Nintendo Wii balance games with stroke patients [31]. A preview of the pro forma logbook is presented in an additional file (see Additional file 2).

#### Secondary outcomes

Secondary outcomes are the objective and subjective balance skills of the participant, assessed at pre- and post-intervention by the study researcher. The **ActiGraph® mobility tracker** assesses movement with a tri-axial accelerometer measuring Local Dynamic Stability (LDS) [32]. The LDS is a non-linear gait stability index quantified by calculating the Lyapunov exponent and has been advocated as an early indicator of the risk of falls [33]. This accelerometer is attached to the patient’s lower back at the level of the third lumbar vertebra and measures the trunk acceleration in medio-lateral, vertical and antero-posterior directions. Participants will be asked to walk as fast as safely possible in a 100-m long corridor (without U-turn). Data analysis will be performed with Matlab (Mathworks, USA) by an external researcher, thereby blinding the outcome analyst towards participants’ group allocation.

The **Falls Efficacy Scale - International Version (FES -I)** is a short, structured, easy to administer questionnaire that measures the level of fear of falling during various social and physical activities (inside and outside the home, whether or not the person actually does the activity). As the balance performance of an elderly person can be translated through the number of falls [10], the FES-I is an indirect indicator for his/her subjective balance abilities. The participants will be asked to fill in the questionnaire themselves. The FES-I shows good validity and reliability and is therefore recommended for research and clinical purposes [34, 35].

### Stratification procedure

Once included, participants will be stratified into four groups according to their computer skills and their balance skills. Computer skills will be assessed on a four-level rating scale that includes *no experience, little experience, some experience* and *plenty of experience*. Participants will then be divided into 2 strata: “no computer skills” identified as *no* or *little experience* and “computer skills” identified as *some* or *plenty of experience*. Berg Balance Scale (BBS) will be used in the assessment of balance capabilities. This clinical test is considered the gold standard for balance assessment [29]. A score < 45 indicates that individuals may be at greater risk of falling [30]. Therefore study participants will be divided into 2 strata: “poor balance capabilities” identified as a BBS score ≤ 44 and “good balance capabilities” identified as a BBS score ≥ 45. The study assessor will assess the computer and balances skills. This stratification is crucial to reduce bias in relation to the primary study question. Firstly, having more computer-experienced participants included in the intervention group should be avoided, and secondly, participants with poor balance skills should be equally distributed.

### Blocked randomisation procedure

An independent investigator with no clinical involvement in the trial has generated the allocation sequence. He created a concealed randomization spreadsheet within these 4 strata using block allocation to assign patients either to conventional self-regulated exercises or self-regulated exergames. Large randomization blocks with a size of 50 participants each were generated on Microsoft Excel (with the Random function). These randomization tables are kept by an independent body responsible for the patient’s therapy planning, namely the therapy secretariat.

### Allocation procedure

After the study researcher measured computer and balances skills for the stratification, he/she will email the therapy secretariat about the results who will then fill in the randomization tables according to the respective strata. Thus the therapy secretariat will be in charge of assigning participants to a group. Patients’ instructions sessions and self-regulated training program will then be planned accordingly and scheduled for the following 10 days of the intervention phase (as per protocol).

### Blinding

Whereas participants and physiotherapists involved in the study will inevitably be aware of the allocation, the outcome assessor (i.e., the study researcher) and the data analyst will be kept blinded to the allocation. The researcher will not be involved in the intervention and the data analyst will be an external professional with no clinical involvement in the trial. Blinding the therapists is generally not possible in exercise trials. To minimize outcome measurement bias, all measurements collected pre- and post-intervention cannot be influenced by therapists providing the intervention nor by the study researcher. The ActiGraph® mobility tracker will measure and save all data directly on the computer in a cryptic version, which will later be analyzed by an external researcher blinded towards participants’ group allocation. Measurements of training volume in the logbook as well as the Falls Efficacy Scale questionnaire are patient-collected data distributing measurement error equally in both groups.

### Sample size

The sample size calculation is based on the observations of a previously performed feasibility study [31] assuming that exergames promoting mobility lead to higher motivation and therefore patients tend to train more often and longer. We assume a statistical power of 0.80 and a medium effect size (d = 0.5). With this effect size of interest in the population, a sample size of 64 subjects per group is necessary to prove any statistical significance. Due to the short observation period of 10 days, less than 10 % drop outs are expected which can be corrected with ITT and multiple imputation of missing data. Alpha is set to 0.05.

### Statistical analysis

Data will be analyzed on an intention-to-treat basis using SPSS version 17.1. Missing outcome data is not expected to be at random and the pattern of missing data not to be monotone. Therefore multiple imputation of missing data will be used. This method improves accuracy of subsequent analyses.

Descriptive analysis will be performed of baseline characteristics (such as sex, age, main diagnostic, MMSE) recorded routinely by the medical team in the patient’s file. The changes of the primary and secondary outcome from study inclusion to discharge will be compared in the two groups with generalized linear models accounting for covariates such as comorbidity.

### Procedure in case of of adverse events

As self-regulated training is already part of the standard services delivered in Walenstadtberg Clinic, patients will not be subjected to more risk or harm by participating in this clinical trial. All included elderly patients are independent walkers (with or without walking aids) and the appropriate exercise level is selected by the physiotherapist according to their balance skills.

In the case of an adverse event (e.g., falls) the in-house protocol is followed as used for all in-patients. The participant is examined by the competent doctor in charge. The physician will then decide on further action to take (e.g., medical care, follow-up consultation, or if necessary, drop-out from the study).

### Ethical considerations

As this clinical study involves human participants, the approval by an ethics committee to conduct the trial is required. Thereby on the 14^th^ of August 2013, the Cantonal Ethics Committee of St. Gallen (Switzerland) has examined the present research project and approved it. The committee’s reference number is EKSG 13/081/1B.

Furthermore patients must agree to participate to the trial by signing a written consent form. Beforehand patients will be fully informed (orally and verbally) about the study goals, procedure and interventions, the risks and benefits, the privacy issues and the possibilities of premature study termination and insurance coverage.

## Results

Recruitment of patients began in July 2014 and is expected to last until the end of 2015. Results analyses are expected in 2016.

## Discussion

According to a database search on PubMed (performed on 06^th^ of June 2015) with the following keywords “exergames”, “serious game”, “elderly people”, “older adults”, “geriatric population” and “randomized controlled trial”, no article could be found on studies comparing any conventional self-regulated exercise program with self-regulated exergames among older post-operative patients. Available studies focus on older adults either suffering from brain injury or cognitive impairment or living in the community.

One of the main characteristics of this trial is its focus on non-supervised exercises. Such self-regulated training has the potential to reach the World Health Organization recommendation for exercise volume in elderly persons over 65 as these can be performed at the person’s own discretion, independently of health care providers. A further focus of this study is on the potential effect of modern game-technology for rehabilitation. We will compare self-regulated exergames with conventional self-regulated exercise programs among elderly post-operative patients. Our hypothesis is that exergames enhance motivation to performed unsupervised exercises thereby leading to increased training volume, and thus to improved balance skills in compared to conventional exercises.

To our knowledge previous studies have focused more on qualitative research than on any clinical outcomes of exergames. The use of game-based technology in elderly users introduces new challenges to the conducting of clinical studies. There is the law of attrition that must be specifically taken into consideration in the evaluation of e-Health trials. Attrition rates are values that indicate participants’ drop out and those might be particularly high in self-help computer-based trials [41]. Special attention must be paid to usage metrics and determinants of attrition in this study with elderly persons, inexperienced in the use of game-based technology. Their measurement and analysis, as well as the characteristics of the subpopulation for which the intervention “works” (in other words, for those who stay in the trial and use it) will be of importance.

A limitation of this study is the short duration of the trial. Although we mentioned in the introduction that to be effective, adherence to training for elderly people must be long lasting, our intervention will last only ten working days. This limitation is due to the hospitalization length of our in-patients. Most patients are referred to our clinic for a 2 to 3 week long rehabilitation program, i.e., 10 to 15 working days. A further limitation of this study is that all included patients were referred for inpatient rehabilitation to Walenstadtberg Rehabilitation Clinic. This clinic provides geriatric post-operative rehabilitation services for the Eastern part of Switzerland. Switzerland is a country with high living standards and life expectancy. This reduces generalizability of the study results to comparable countries only.

## Additional files

Additional file 1:
**Conventional self-regulated exercise programs.** Illustrations and written instructions for each self-regulated exercise. A trained physiotherapist teaches the customized program and a printed handout is given to the patient. (DOCX 1587 kb) Additional file 2:
**Self-regulated training logbook.** Preview of one page of the pro forma logbook distributed to every study participant. The patient should adequately fill one page for each working day, according to the performed self-regulated training. (DOCX 24 kb)
