# Supplementary material for: Are exergames promoting mobility an attractive alternative to conventional self-regulated exercises for elderly people in a rehabilitation setting? Study protocol of a randomized controlled trial
Source: BMC Geriatr. 2015 Sep 7;15:108. doi: 10.1186/s12877-015-0106-0 (PMC4562105; doi:10.1186/s12877-015-0106-0)
Supplement: Additional file 1: — Conventional self-regulated exercise programs. Illustrations and written instructions for each self-regulated exercise. A trained physiotherapist teaches the customized program and a printed handout is given to the patient. (DOCX 1587 kb) [file 12877_2015_106_MOESM1_ESM.docx]

**Additional file 1: Conventional self-regulated exercise programs (handouts)**

| **Conventional self-regulated exercises Level 1**  **BBS<45, Exercises in sitting position** |
| --- |

Name: Surname: Birthdate:

**Exercise 1:**

| 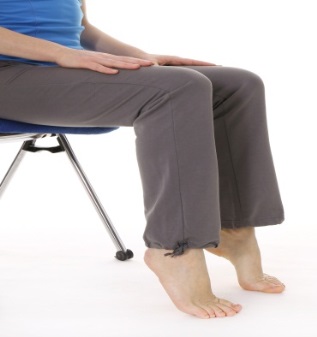 | Sitting upright, lift heels and tip toes alternatively.  Repetitions: 3 X 12 | 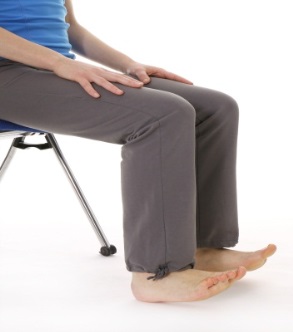 |
| --- | --- | --- |

**Exercise 2:**

|  | Sitting upright, move foot forwards and backwards alternatively („wipe“)  Repetitions: until tired |  |
| --- | --- | --- |

**Exercise 3:**

| 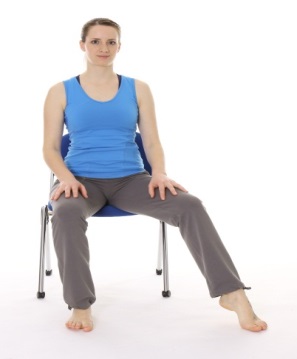 | Sitting upright, reach with the right foot as far as possible to the right. The same with left foot to the left.  Repetitions: 2 x 10 (for each side) | 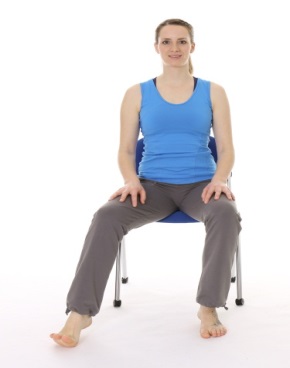 |
| --- | --- | --- |

**Exercise 4:**

|  | Sitting upright, stand up and sit down with the least possible arm support.  Repetitions: 3 x 12 |  |
| --- | --- | --- |

**Exercise 5 :**

| 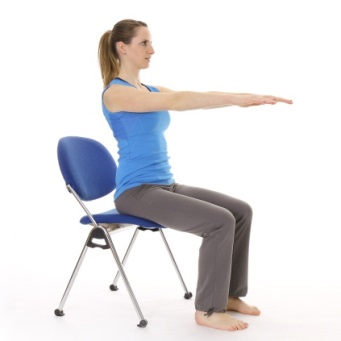 | Sitting upright, stretch arms forwards and move trunk as far as possible forward.  Repetitions: 3 x 12 | 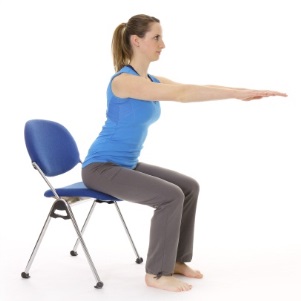 |
| --- | --- | --- |

**Exercise 6 :**

| 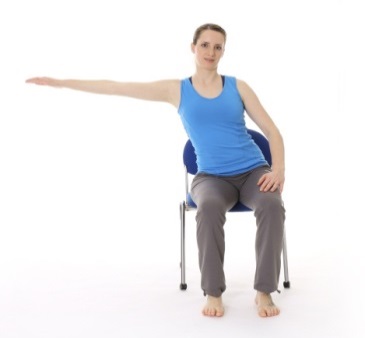 | Sitting upright, reach with the stretched arm as far as possible to one’s side.  Repetitions: 2 x 10 (for each side) | 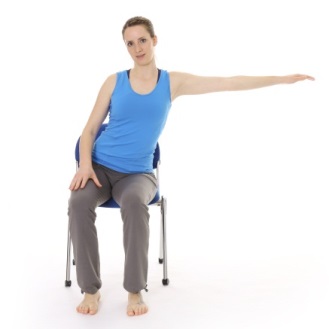 |
| --- | --- | --- |

**Exercise 7:**

| 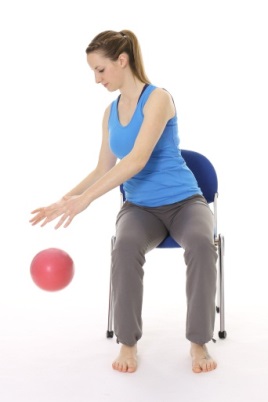 | 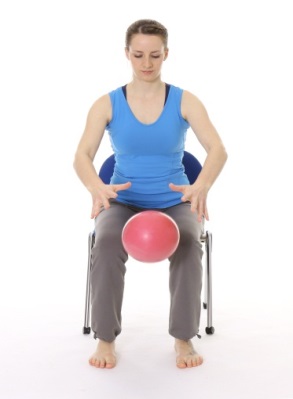 | 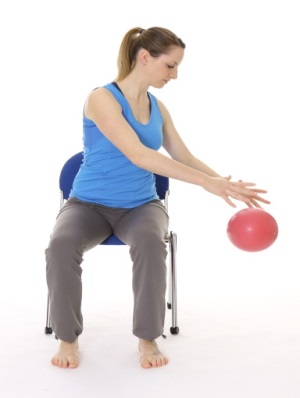 |
| --- | --- | --- |

Sitting upright, dribble the ball with both hands in the front and on the sides.

Repetitions: 2 x 10 in each position

| **Conventional self-regulated exercises Level 2**  **45 ≤BBS <56, Exercises in standing** |
| --- |

Name: Surname: Birthdate:

**Exercise 1:**

|  | Alternate standing on toes and standing on heels.  Repetitions: 3 x 10 |  |
| --- | --- | --- |

**Exercise 2:**

|  | In step position, keep arms crossed and turn the head alternatively to the right to see behind the shoulder and to the left.  Repetitions: 3 x 10 |  |
| --- | --- | --- |

**Exercise 3:**

|  | Stand on a line with L toes as close as possible to the R heel.  Keep position as long as possible. Then alternate feet. |  |
| --- | --- | --- |

**Exercise 4:**

|  | Touch each red spot with one foot. Then same with one foot.  Repetitions: 3 x with each foot |  |
| --- | --- | --- |

**Exercise 5:**

|  | In step position, throw a ball from one hand to the other.  Repetitions: 2 x 10 |  |
| --- | --- | --- |

| **Conventional self-regulated exercises Level 3**  **BBS =56, Exercises in walking** |
| --- |

Name: Surname: Birthdate:

**Exercise 1:**

|  | Walk forward on tip toes and backwards on heels.  Repetitions: 10 x 5 meters |  |
| --- | --- | --- |

**Exercise 2:**

|  | Walk on a line with toes as close as possible to heel.  Repetitions: 10 x 5 meters |  |
| --- | --- | --- |

**Exercise 3:**

|  | Arms crossed, step sideways on a line.  Repetitions: 10 x back and forth |  |
| --- | --- | --- |

**Exercise 4:**

|  | Arms crossed, do cross steps on a line.  Repetitions: 10 x back and forth |  |
| --- | --- | --- |

**Exercise5:**

|  | In walking, throw a ball from one hand to the other.  Repetitions: 5 x 5 meters |  |
| --- | --- | --- |
