# Supplementary material for: Are exergames promoting mobility an attractive alternative to conventional self-regulated exercises for elderly people in a rehabilitation setting? Study protocol of a randomized controlled trial
Source: BMC Geriatr. 2015 Sep 7;15:108. doi: 10.1186/s12877-015-0106-0 (PMC4562105; doi:10.1186/s12877-015-0106-0)
Supplement: Additional file 2: — Self-regulated training logbook. Preview of one page of the pro forma logbook distributed to every study participant. The patient should adequately fill one page for each working day, according to the performed self-regulated training. (DOCX 24 kb) [file 12877_2015_106_MOESM2_ESM.docx]

**Additional file 2: Self-regulated training logbook**

…….**/**…….**/ 2015**

**Date of today:**

**How many times have you performed the self-regulated training today?**

|  | **Length (in minutes)** | **Comments, problems encountered** |
| --- | --- | --- |
| 1. **Self-regulated training** |  |  |
| 1. **Self-regulated training** |  |  |
| 1. **Self-regulated training** |  |  |
| 1. **Self-regulated training** |  |  |

**ca. minutes**

**For how many minutes did you specifically go for walking today?**

**How motivated were you for the self-regulated training today?**

**□no motivation □low motivation □some motivation □high motivation □huge motivation**

**Did you have fun during the self-regulated training today?**

**□ no fun □ a little fun □ some fun □ lots of fun □ extremely fun**
